# Preparation of Quercetin/Copper Nanoparticles and Their Preservation Performance on *Shine Muscat* Grapes

**DOI:** 10.3390/molecules30071438

**Published:** 2025-03-24

**Authors:** Kundian Che, Zhanjun Chen, Luo Weng, Baogang Zhou, Wei Gao, Ran Liu, Jialin Yang, Haoyuan Luo, Wenzhong Hu

**Affiliations:** 1 College of Life Science, Zhuhai College of Science and Technology, Zhuhai 519040, China; che1285919426@163.com (K.C.); zhanjun_chen@outlook.com (Z.C.); wlabwengluo@163.com (L.W.); 18043217471@163.com (B.Z.); 15636318098@163.com (W.G.); liur0050@gmail.com (R.L.); jialiny23@163.com (J.Y.); luohaoyuan2021@163.com (H.L.); 2 College of Life Science, Jilin University, Changchun 130015, China

**Keywords:** metal/phenolic networks, antibacterial, antioxidant, fruit preservation technology, *Shine Muscat* grapes

## Abstract

Deterioration in fruits represent a significant challenge to food safety, which has prompted our investigation into sustainable fruit preservation technologies. This paper presents the synthesis of quercetin/copper nanoparticles (QC NPs) and their application in the preservation of *Shine Muscat* grapes. The QC NPs, prepared through quercetin/copper complexation, exhibited stability with a particle size of 79.4 ± 3.2 nm and a zeta potential of −34.00 ± 4.98 mV. The nanoparticles exhibited robust antioxidant activity and 100% bactericidal effect against *E. coli* and *S. aureus* at 0.05 mg/mL, thereby underscoring their potential for use in fruit preservation. The application of a sodium alginate (SA) + QC NP coating to *Shine Muscat* grapes resulted in an 8.08% reduction in weight loss in comparison to the control, which exhibited a 10.40% reduction. The coating maintained firmness and preserved titratable acid content, thereby extending the storage life of the grapes. These findings position QC NPs as a promising material in eco-friendly and effective fruit preservation, and offer a viable solution to postharvest fruit preservation.

## 1. Introduction

Conducted in 1988, the cultivar ‘*Shine Muscat’* was derived from a cross between Akitsu-21 (*Vitis labruscana* Bailey) and ‘Hakunan’ (*Vitis vinifera* L.). Akitsu-21 is a hybrid of ‘Steuben’ (*Vitis labruscana*) × ‘Muscat of Alexandria’ (*Vitis vinifera*) [1]. The fruit is characterized by its distinct rose aroma coupled with an excellent sweetness and taste profile, low acidity, and a range of other favorable attributes [2]. These attributes have contributed to its popularity among consumers in East Asia, China, Japan, and other countries. As a result, the fruit is now widely cultivated in China, where it is undergoing a period of rapid development [3]. It is noteworthy that China loses over 20% of its postharvest grapes to rot each year, which has an adverse effect on the development of industries related to grapes [4]. The *Shine Muscat* grape is a non-climacteric fruit, meaning that it can only be picked after reaching full ripeness on the plant, as it lacks the ability to ripen independently. Following harvest, *Shine Muscat* grapes are susceptible to moisture transfer, browning, degranulation, and rotting during storage and transportation due to their characteristic thin skin, soft texture, and juiciness, which provide conducive conditions for the growth and reproduction of bacteria [5]. Consequently, the pursuit of environmentally benign and effective techniques for the preservation of fruits remain a pivotal area of investigation.

Copper-based systems have emerged as promising candidates for food preservation due to their inherent dual functionality as both an antioxidant and antimicrobial agent. Green-synthesized copper nanoparticles (Cu NPs) exhibit broad-spectrum antimicrobial activity through mechanisms involving reactive oxygen species (ROS) generation and membrane depolarization, while plant-derived polyphenols in these systems retain their intrinsic antioxidant capacity [6]. Notably, catechin/copper complexes demonstrate retained free radical scavenging activity, coupled with enhanced antimicrobial efficacy, through synergistic interactions between polyphenolic ligands and copper ions [7]. The biocidal action of copper species is attributed to nascent Cu^2+^ ions released from nanoparticle surfaces, which induce lipid peroxidation, protein oxidation, and DNA degradation in microbial cells [8]. These properties collectively address the requirements for postharvest protection while mitigating environmental impact.

Quercetin is a naturally occurring polyhydroxy dietary flavonoid that is widely found in vegetables and fruits and has been demonstrated to possess antioxidant and antibacterial activity [9]. Nevertheless, the restricted antibacterial activity of quercetin as a natural plant extract necessitates the utilization of exceedingly high concentrations to attain the desired antibacterial activity, which may potentially impair the flavor and quality of the food product. Additionally, the poor water solubility of quercetin, which impedes its dispersion in polymer matrices, further constrains its application in the food industry [10].

In recent years, metal/phenolic networks formed by coordination interactions between multivalent metal ions and polyphenols have received much attention due to their synergistic effect with properties of metal ions and polyphenols, as well as their effective dispersion in water and good bioavailability [11]. Yueying Xu et al. used dihydroartemisinin complexed with Fe_2_O_3_ to form nanoparticles that could effectively inhibit the growth of *E. coli* and *S. aureus* [12]. Rongxin Yu et al. assembled nanoparticles using (−)-epigallocatechin gallate (EGCG) with copper ions and diethyldithiocarbamate. Their findings demonstrated a notable inhibitory effect on methicillin-resistant *S. aureus*, and the nanoparticles were confirmed to exhibit minimal toxicity across a range of models, from cellular to blood, zebrafish, nematode, and mouse [13]. Haijing Qu et al. prepared a quercetin and tannic acid nanoparticle coordinated with trivalent iron (Fe^3+^), which has a strong antioxidant capacity. This nanoparticle is capable of purging ROS, repairing mitochondrial damage, and upregulating Nrf2/HO-1, thereby effectively alleviating oxidative stress and regulating the redox balance. Additionally, this nanoparticle can induce M1 macrophage polarization in an anti-inflammatory M2 subtype, which improves the inflammatory microenvironment [14].

The experimental design incorporated SA as the polymeric matrix due to its generally recognized as safe (GRAS) status and exceptional oxygen barrier properties. Potassium sorbate was selected as the positive control based on its established efficacy as a broad-spectrum food preservative. This conventional preservative provides a benchmark for the evaluation of the preservation effects of QC NPs within an SA matrix.

In light of the aforementioned issues, this study utilized quercetin complexed with copper ions to form nanoparticles (QC NPs) that were then dispersed in sodium alginate to create a coating to preserve the freshness of *Shine Muscat* grapes. This preservation coating exhibited antioxidant properties and demonstrated the capacity to effectively inhibit the growth of pathogenic microorganisms. Furthermore, it extended the storage period of *Shine Muscat* grapes.

## 2. Results and Discussion

### 2.1. Characterization of QC NPs

#### 2.1.1. Particle Size, Zeta Potential, and PDI of QC NPs

**Figure 1:**
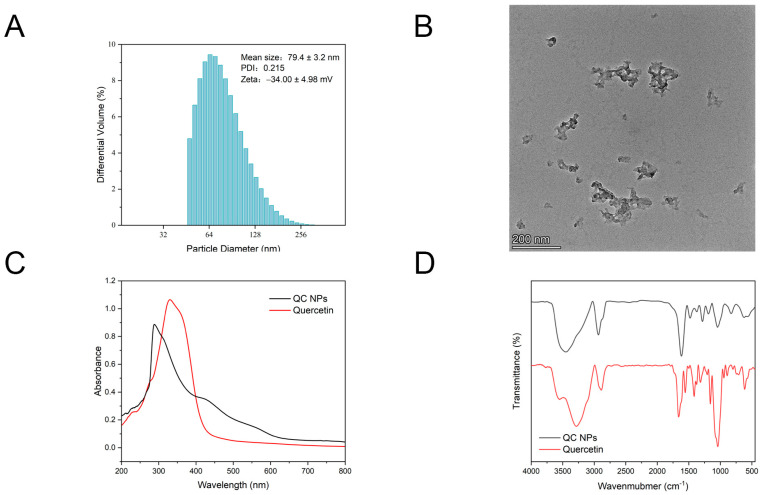
(**A**) Particle size, zeta potential, and PDI of QC NPs, (**B**) TEM micrographs of QC NPs, (**C**) UV–vis spectroscopy of quercetin and QC NPs, and (**D**) FTIR spectroscopy of quercetin and QC NPs.

According to Figure 1A,B, the particle size of the QC NPs was 79.4 ± 3.2 nm, which can be also observed by TEM. The findings are consistent with the fundamental characteristics of nanomaterials ranging from 1 to 100 nm. Colloidal stability exhibits a direct correlation with absolute zeta potential magnitude, where elevated values impart colloidal stabilization through electrostatic repulsion forces that effectively prevent particle aggregation. Conversely, lower zeta potential magnitudes result in insufficient interparticle repulsion, leading to colloidal instability through aggregation phenomena. The zeta potential value of the QC NPs is −34.00 ± 4.98 mV. The absolute value of the zeta potential is greater than that of several quercetin microemulsions and nanoparticles [15,16]. This finding suggests that QC NPs can maintain good dispersion stability in a solution by electrostatic interaction. In addition, PDI represents the uniformity of the particle size distribution; a smaller PDI represents a more uniform distribution of molecular weight. The PDI value of the QC NPs was lower than 0.3, which represents the homogeneous or monodispersed particle population of the system [16].

#### 2.1.2. UV–Vis Spectroscopy and FTIR Spectroscopy of QC NPs

According to Figure 1C, the characteristic absorption peak of quercetin occurs at approximately 370 nm, which can be attributed to the presence of aromatic rings and conjugated double bonds. The characteristic absorption peak of the QC NPs occurs at approximately 289 nm. This spectral blue shift indicates the occurrence of a coordination interaction between quercetin and copper.

According to Figure 1D, the broad absorption band at 3200–3500 cm^−1^ corresponds to -OH stretching vibrations [17], while the peaks at ~2900 cm^−1^ and ~1450 cm^−1^ are assigned to CH stretching and bending modes, respectively. Distinctive vibrations include C=O stretching of the ketone group (~1650 cm^−1^) and CO stretching of the ether group (~1100 cm^−1^) [14]. The split peaks near 750 cm^−1^, characteristic of out-of-plane CH bending in the ortho–dihydroxy-substituted benzene ring, disappear in the QC NPs, indicating coordination between copper ions and the catechol–hydroxyl groups of quercetin.

#### 2.1.3. XPS of QC NPs and Quercetin

**Figure 2:**
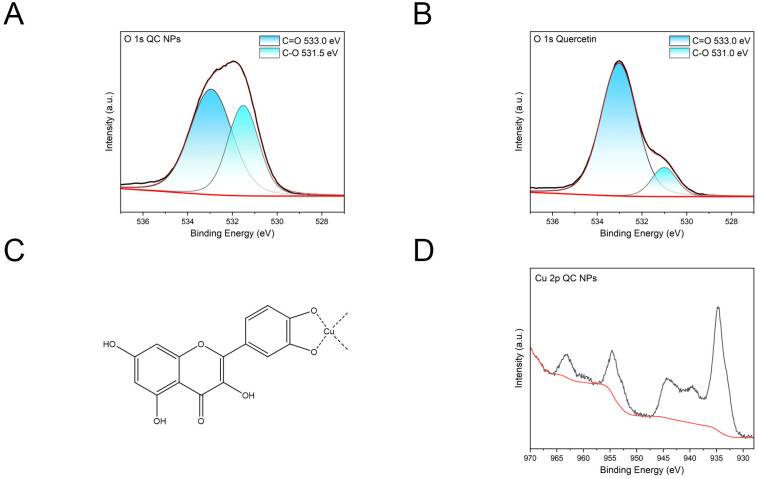
(**A**) O 1s XPS spectral deconvolution of QC NPs, (**B**) O 1s XPS spectral deconvolution of quercetin, (**C**) proposed coordination architecture of QC NPs, and (**D**) Cu 2p XPS fingerprint of QC NPs. The red line in the picture is the Background Intensity (a.u.).

According to Figure 2A,B, the O 1s spectral deconvolution of quercetin revealed two distinct binding energy components at 531.0 eV and 533.0 eV. The higher-energy component (533.0 eV) corresponds to the C=O group, showing negligible low-energy shift compared to the characteristic ligand coordination shifts (0.3–0.8 eV) observed in the QC NPs. This absence of chemical shift indicates non-participation of the carbonyl group in copper ion coordination. Conversely, the CO-associated peak at 531.0 eV demonstrates a 0.5 eV positive binding energy shift relative to the corresponding peak (531.5 eV) of the QC NPs. This energetic displacement suggests reduced electron density on hydroxyl oxygen atoms due to coordination with copper ions. Collectively, these observations substantiate that QC NPs formation involves selective coordination between copper ions and quercetin’s hydroxyl groups while the carbonyl moiety remains non-coordinated, consistent with the proposed structure in Figure 2C.

According to Figure 2D, the diagnostic features of Cu^2+^ comprise a characteristic peak at 963 eV with characteristic shake-up satellite signatures spanning 939–945 eV. This spectral fingerprint provides conclusive evidence of both the effective formation of QC NPs and the retention of divalent copper species within QC NPs.

### 2.2. Pharmacological Activity of QC NPs

#### 2.2.1. The Antioxidant Activity and Antibacterial Activity of QC NPs

**Figure 3:**
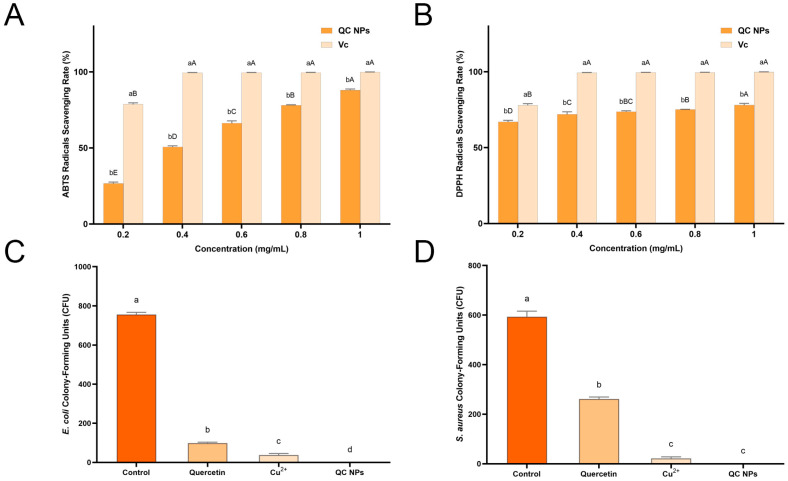
(**A**) ABTS radical scavenging rate of ascorbic acid (Vc) and QC NPs, (**B**) DPPH radical scavenging rate of Vc and QC NPs, (**C**) *E. coli* CFU among treatment groups, and (**D**) *S. aureus* CFU among treatment groups. Data are expressed as mean ± SD (*n* = 3). Statistical analysis was performed using two-way ANOVA with Bonferroni test. Significant differences are denoted by lowercase and capital letters (a, b, c, d, A, B, C, D, E; *p* < 0.05). Quercetin concentration was 0.047 mg/mL and Cu^2+^ concentration was 0.003 mg/mL, both of which were consistent with their concentrations in 0.05 mg/mL QC NPs.

Oxidative processes cause the chemical degradation of the nutrients present in fruits, including ascorbic acid (vitamin C, V_C_) and carotenoids. This results in a significant alteration to the appearance and flavor of fruits, which are perceived as less appetizing as a result. Therefore, the antioxidant efficacy of QC NPs was evaluated using ABTS and DPPH radical scavenging assays, commonly employed to assess the antioxidant potential of compounds. (Figure 3A,B) The results of the ABTS radical scavenging assay demonstrate that the ABTS radical scavenging rate of the QC NPs exhibits a notable increase with an elevation in the concentration of QC NPs from 26% to 88% (EC_50_ = 0.3814 mg/mL). In the DPPH radical scavenging assay, the QC NPs demonstrate notable antioxidant activity, with the DPPH radical scavenging rate increasing from 67% to 78% (EC_50_ = 0.0219 mg/mL) with an increase in QC NP concentration. Notably, QC NPs outperformed natural antioxidants such as bee pollen nano/microparticles (EC_50_ = 5.4 ± 0.07 mg/mL) and mango kernel marc extracts (EC_50_ = 0.20–0.22 mg/mL) in efficacy [18,19]. These concentration-dependent profiles confirm preserved antioxidant functionality of quercetin’s phenolic hydroxyl groups despite their participation in copper coordination, which is of significant value for the preservation of fruits and can effectively mitigate oxidation and degradation in fruits, thereby prolonging their shelf life [20,21].

**Figure 4:**
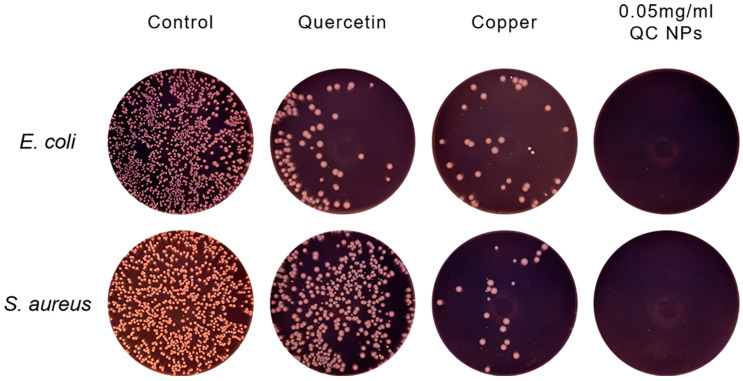
Representative photographs of *E. coli* and *S. aureus* colonies formed on agar plates among treatment groups.

Antibacterial activity can reduce or prevent food spoilage caused by harmful pathogens; the antibacterial activity of the QC NPs was evaluated against Gram-negative bacteria (*E. coli*) and Gram-positive bacteria (*S. aureus*) (Figure 3C,D and Figure 4). At 0.05 mg/mL (50 µg/mL), the QC NPs demonstrate complete bacterial eradication (99.99% reduction) of both pathogens, outperforming not only individual quercetin and Cu^2+^ components, but also the reported antibacterial efficacy of TA-Fe/Cu NPs (200 µg/mL) [22]. This study demonstrates that QC NPs exhibit remarkable bacteriostatic activity.

#### 2.2.2. Mechanism of Antibacterial Activity by QC NPs

**Figure 5:**
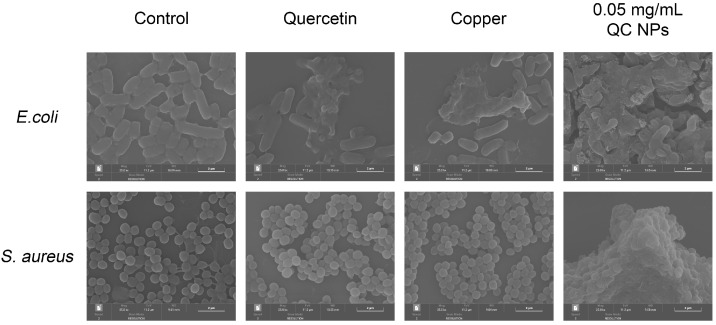
SEM micrographs of *E. coli* and *S. aureus* among treatment groups.

**Figure 6:**
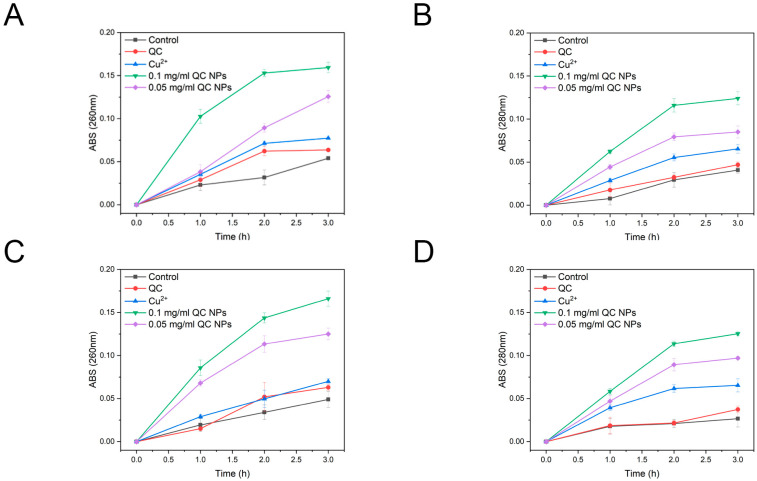
The leakage of nucleic acids and proteins from *E. coli* and *S. aureus* among the treatment groups. *E. coli* nucleic acids (**A**), *E. coli* proteins (**B**), *S. aureus* nucleic acids (**C**), and *S. aureus* proteins (**D**). Data are expressed as mean ± SD (*n* = 3). Statistical analysis was performed using two-way ANOVA with the Bonferroni test. The quercetin concentration was 0.047 mg/mL and Cu^2+^ concentration was 0.003 mg/mL, both of which were consistent with their concentrations in 0.05 mg/mL QC NPs.

According to Figure 5, SEM micrographs reveal pronounced membrane disruption in both *E. coli* and *S. aureus*, as evidenced by their collapsed cellular morphology and cytoplasmic content leakage. Mechanistic studies through nucleic acid and protein leakage assays in Figure 6 demonstrate concentration-dependent and time-dependent release kinetics of intracellular components. Notably, distinct leakage patterns exhibit significantly higher absorbance at 260 nm (nucleic acid-specific) than at 280 nm (protein-specific) resulting from the following two factors:

Extinction coefficient difference: nucleic acids display stronger UV absorption owing to π-conjugated aromatic systems in purine/pyrimidine bases, yielding enhanced detection sensitivity (1 OD_260_ = 50 μg/mL dsDNA vs. 1 OD_280_ = 1 mg/mL protein).

Size-dependent permeabilization: the lower molecular mass of nucleic acids facilitates their preferential efflux through nanoparticle-generated membrane pores.

These findings collectively demonstrate that QC NPs compromise bacterial membrane integrity, inducing content leakage.

**Figure 7:**
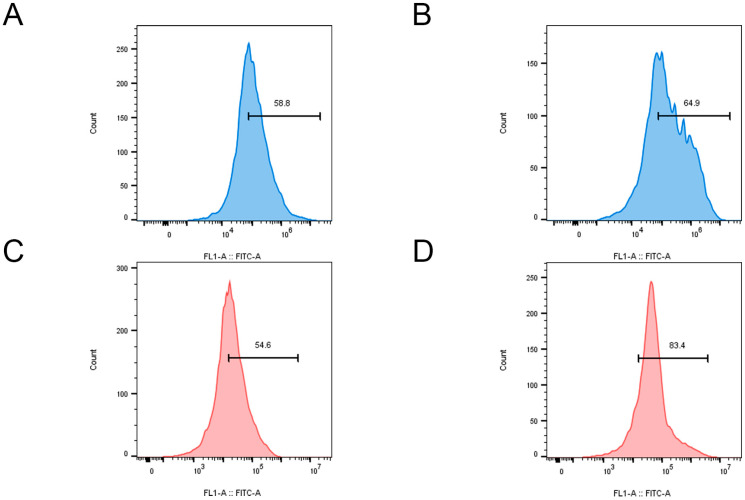
ROS content (fluorescence intensity) of *E. coli* and *S. aureus* in control and QC NP groups. *E. coli* in control group (**A**), *E. coli* in QC NP group (**B**), *S. aureus* in control group (**C**), and *S. aureus* in QC NP group (**D**). QC NP concentration was 0.05 mg/mL.

Copper ions, as a redox-active metal, have been demonstrated to generate substantial quantities of ROS within cells through the Fenton reaction [23]. High levels of ROS attack the cell membrane and induce cell death [24]. The fluorescent probe 2′,7′-dichlorofluorescin diacetate (DCFH-DA) and flow cytometry were employed to detect the production of ROS within the bacteria, with the fluorescence intensity serving as an indicator of ROS levels in bacterial cells. In *E. coli*, treatment with QC NPs resulted in a significant increase in fluorescence intensity, indicating a substantial rise in intracellular ROS (Figure 7A,B). This increase in ROS is crucial for the disruption of bacterial cellular functions, which ultimately leads to membrane damage and potential cell death. Similarly, in the case of *S. aureus*, the fluorescence intensity also increased markedly upon treatment with QC NPs (Figure 7C,D). These findings are consistent with the current understanding that an elevated ROS level can overwhelm bacterial antioxidant defenses, leading to oxidative damage and cell death. The data on ROS from both *E. coli* and *S. aureus* provide evidence for the antimicrobial mechanism of QC NPs.

#### 2.2.3. Biofilm Degradation Activity of QC NPs

**Figure 8:**
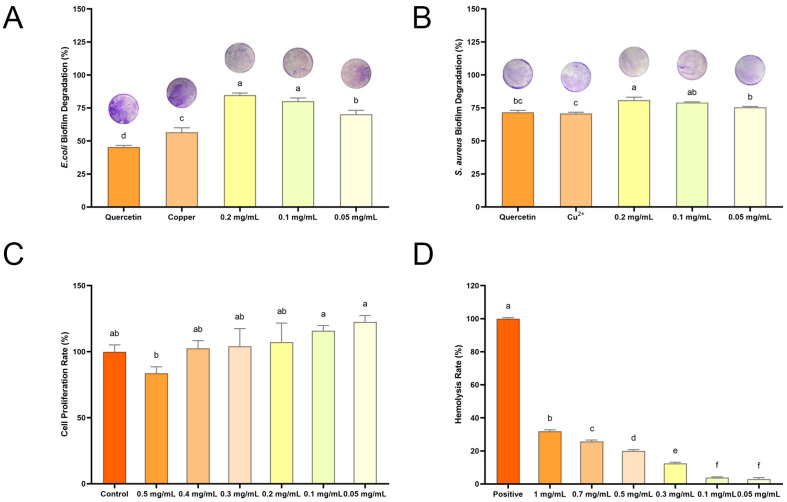
(**A**) Biofilm degradation rate of *E. coli* among treatment groups, (**B**) biofilm degradation rate of *S. aureus* among treatment groups, (**C**) cell proliferation rate among treatment groups, and (**D**) hemolysis rate among treatment groups. Data are expressed as mean ± SD (*n* = 3). Statistical analysis was performed using two-way ANOVA with Bonferroni test. Significant differences are denoted by lowercase letters (a, b, c, d, e, f and *p* < 0.05). Quercetin concentration was 0.047 mg/mL and Cu^2+^ concentration was 0.003 mg/mL, both of which were consistent with their concentrations in 0.05 mg/mL QC NPs.

ROS can damage biofilms by directly reacting with the extracellular polymeric substances matrix and the microorganisms that are embedded within the biofilm [25]. Given that QC NPs are capable of generating a substantial amount of ROS in bacterial cells, the influence of their inhibition ability on the rate of bacterial biofilm formation was further explored by a crystal violet staining experiment. Figure 8A illustrates that, even at the lowest concentration tested (0.05 mg/mL), QC NPs induce over 70% biofilm degradation in *E. coli*, thereby demonstrating the potency of their degradation activity against established biofilms. Similarly, Figure 8B illustrates that QC NPs effectively degrade *S. aureus* biofilms in a concentration-dependent manner. At concentrations of 0.1 mg/mL and 0.2 mg/mL, QC NPs achieve over 80% biofilm degradation. Furthermore, these figures demonstrate the concentration-dependent effect of QC NPs on biofilm degradation, with significant implications for their potential use in antimicrobial strategies.

### 2.3. Biosafety of QC NPs

The biosafety of QC NPs was evaluated with a cytotoxicity assay and a hemolysis assay. The cytotoxicity assay was conducted using a Cell Counting Kit-8 (Beyotime, Shanghai, China) with the L929 cell as the cell model and the cell proliferation rate serving as an indicator for cytotoxicity analysis (Figure 8C). The graph illustrates a concentration-dependent decline in cell proliferation rate. Notably, low concentrations of QC NPs (0.05 mg/mL to 0.4 mg/mL) have the potential to surpass the control group (>100%) in terms of cell proliferation rate. From a biosafety perspective, these doses are deemed to be relatively safe. This suggests that QC NPs do not exert a detrimental impact on cell proliferation within a specific concentration range, indicating a low level of cytotoxicity.

The hemolysis assay employs the hemolysis rate as an analytical index, with the hemolysis rate determined by measuring the absorbance value of the supernatant (Figure 8D). The graph illustrates a concentration-dependent increase in hemolysis, with low concentrations of QC NPs (0.05 mg/mL to 0.1 mg/mL) exhibiting minimal hemolysis, less than 5%. This suggests that these doses are highly safe from a biosafety perspective. It indicates that QC NPs, within a certain concentration range, do not adversely affect red blood cell integrity, which is a key consideration for their potential use in biological systems. As the concentration of QC NPs increases, there is a corresponding rise in the hemolysis rate, with the highest concentration tested (1 mg/mL) demonstrating a more pronounced effect.

### 2.4. Coating Preservation of Shine Muscat Grapes

#### 2.4.1. The Appearance of *Shine Muscat* Grapes

**Figure 9:**
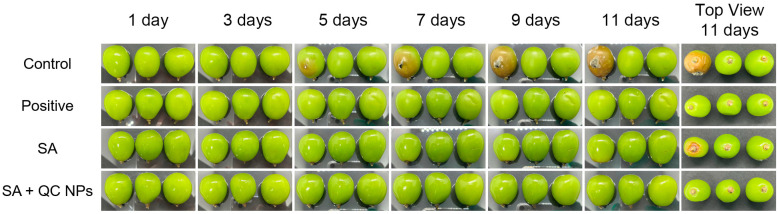
Photographs of *Shine Muscat* grapes among treatment groups during storage.

Appearance is a significant indicator in the measurement of the quality of fruits, as it allows consumers to make rapid and intuitive assessments [5]. The *Shine Muscat* grape is susceptible to browning when stored for extended periods, which is an outward indicator of spoilage [26]. This characteristic can be identified through apparent picture analysis (Figure 9). The control group exhibited visible browning with decay on day 5. In contrast, the SA group demonstrated browning on day 9 without any accompanying decay. The positive group did not display either browning or rotting, but exhibited multiple brown spots on the epidermis, suggesting compromised epidermal integrity. In comparison, the SA + QC NP group retained its initial appearance until day 11. The top view on day 11 corroborated the presence of rot in the control and SA groups. Additionally, it was observed that the grapes in all groups exhibited a range of epidermal folds.

#### 2.4.2. The Weight Loss and Firmness of *Shine Muscat* Grapes

**Figure 10:**
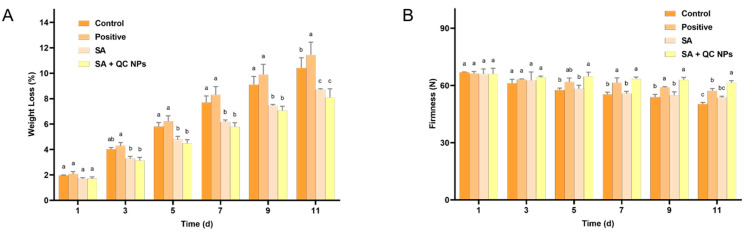
(**A**) Weight loss of *Shine Muscat* grapes among treatment groups and (**B**) firmness of *Shine Muscat* grapes among treatment groups. Data are expressed as mean ± SD (*n* = 3). Statistical analysis was performed using two-way ANOVA with Bonferroni test. Significant differences are denoted by lowercase letters (a, b, c, and *p* < 0.05).

In storage, weight loss is an invaluable indicator of dehydration and deterioration in quality of fruits and vegetables. The primary cause of weight loss is the loss of water through transpiration [27]. A preservation coating can serve as a physical barrier, preventing the loss of water from fruits and thereby reducing weight loss [28]. According to Figure 10A, the weight loss of each group of grapes exhibited a gradual decline over time. On day 11 of storage, the rate of weight loss was found to be positive (11.42%) > control (10.40%) > SA (8.71%) > SA + QC (8.08%), where the uncoated group demonstrated a significantly higher rate of weight loss than the coated group. The results show that the preservation coating method is the most effective treatment to minimize weight loss, thereby preserving the freshness and quality of *Shine Muscat* grapes.

The firmness of a fruit is dependent upon the cellular structure of the fruit, which can be indicative of its freshness, ripeness, and age [29]. It is typical for fruits to undergo a ripening and ageing process when stored for an extended period of time. This process results in a loss of internal water and the degradation of cell walls by enzymes, which leads to a loss of firmness [30,31]. According to Figure 10B, all experimental groups exhibited a progressive loss of firmness attributable to postharvest metabolic activity and cellular senescence. However, the rate and extent of this decline differed significantly between the treatment groups. The control group exhibited the most pronounced reduction in firmness, indicating a rapid loss of structural integrity associated with cell wall degradation and dehydration. In contrast, the SA + QC NP group exhibited the slowest loss of firmness rate, retaining the highest firmness values throughout the storage period. These results demonstrate that the SA + QC NP coating effectively maintains cellular turgor pressure through moisture retention while preventing structural damage induced by microbial colonization. The enhanced preservation of firmness by the SA + QC NP coating primarily arises from the dual mechanism of effective microbial growth suppression coupled with a nanocomposite barrier that minimizes moisture loss.

#### 2.4.3. The Titratable Acid (TA) Content of *Shine Muscat* Grapes

**Figure 11:**
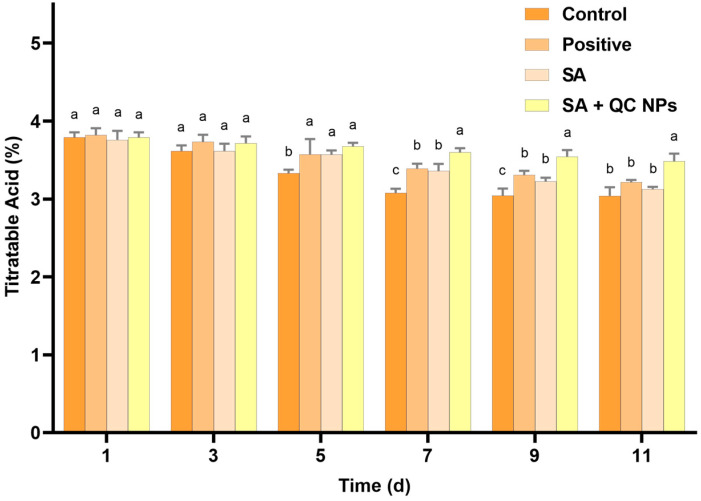
TA of *Shine Muscat* grapes among treatment groups. Data are expressed as mean ± SD (*n* = 3). Statistical analysis was performed using two-way ANOVA with Bonferroni test. Significant differences are denoted by lowercase letters (a, b, c, and *p* < 0.05).

Titratable acid (TA) is a pivotal parameter in the evaluation of grape quality, as it exerts a substantial influence on organoleptic characteristics, including taste and preservation status. TA, which is primarily constituted by organic acids such as tartaric and malic acid, not only contributes to the distinctive flavor profile of grapes but also serves as a crucial substrate in respiratory metabolism [32,33,34]. According to Figure 11, the TA content demonstrates a gradual decline across all treatment groups, which is consistent with the natural metabolic processes that occur during fruit storage. The control group exhibited the most rapid decrease in TA content. In contrast, the SA + QC NP group exhibited a markedly slower rate of decline in TA content compared to the other groups. These observations indicate that the SA + QC NP coating effectively retards the respiration and metabolic processes of the grapes, thereby preserving the organic acids and contributing to overall quality maintenance.

## 3. Materials and Methods

### 3.1. Materials and Reagents

Quercetin, cupric chloride dihydrate (CuCl_2_·2H_2_O), glutaraldehyde and paraformaldehyde were obtained from Macklin Co., Ltd. (Shanghai, China). Potassium persulfate and crystal violet were obtained from Xilong Co., Ltd. (Shantou, China). ABTS diammonium salt, DPPH, and Triton X-100 were obtained from Yuanye Co., Ltd. (Shanghai, China). Nutrient broth and TTC nutrient agar were obtained from HuanKai Co., Ltd. (Guangzhou, China). *E. coli* and *S. aureus* were obtained from Guangdong Microbial Culture Collection Center (GDMCC). L929 was obtained from Procell Co., Ltd. (Taoyuan, China). Solvents, including methanol, ethanol, and acetic acid, were analytical grade and used without further purification.

### 3.2. Preparation and Characterization of QC NPs

Preparation of the QC NPs was optimized using the following synthetic method [35]: A total of 6 mL quercetin solution (0.03 M in 0.1 M NaOH) was mixed with 32.2 mL deionized water. Then, 1.8 mL CuCl₂ solution (0.1 M in deionized water) was slowly added and stirred, the molar ratio of quercetin––to–copper ions being 1:1. The pH of the mixture was then adjusted 7.6 with 0.1 M HCl solution and 0.1 M NaOH solution and stirred for 30 min. The product was separated by centrifugation at 12,000 rpm, washed three times with deionized water, and resuspended in deionized water. Thus, the QC NPs were prepared.

Particle size, zeta potential, and polymer dispersity index (PDI) of the QC NPs were obtained using a Zeta/Nano Particle Analyzer (NanoPlus-3, Micromeritics, Norcross, GA, USA). The morphology of the QC–NPs was examined using a Transmission Electron Microscope (Talos F200S, Thermo Fisher Scientific, Waltham, MA, USA). The ultraviolet–visible (UV–vis) absorption spectrum and Fourier transform infrared (FTIR) spectrum of the QC NPs were obtained using a UV–vis Spectrophotometer (UV-2600, Shimadzu, Tokyo, Japan) and an FTIR Spectrometer (IR Prestige-21, Shimadzu, Japan). The X-ray photoelectron spectroscopy (XPS) of the QC NPs and quercetin was examined using a X-ray Photoelectron Spectrometer (K-ALPHA, Thermo Fisher Scientific, USA). The concentration of copper ions in the QC NPs was determined by Atomic Absorption Spectrophotometer. (AA-7000, Shimadzu, Japan).

### 3.3. Analysis of Antioxidant Activities of QC NPs

#### 3.3.1. ABTS Radical Scavenging Activity

The ABTS radical scavenging assay method was adapted based on its use in reference number [36]. ABTS (7.4 mM) was mixed with 2.45 mM potassium persulfate for 12–24 h in the dark and the absorbance of the reaction mixture was adjusted with ethanol to 0.70  ±  0.02 at 732 nm of ABTS solution. An aliquot (20 µL) of a sample was reacted with 180 µL ABTS (A732 nm  =  0.70  ±  0.02) for one minute and the absorbance of the reaction mixture was measured at 732 nm. Percentage of antioxidant activity was compared against the positive control, a standard solution of ascorbic acid, and calculated using the following equation [37]:

(1) $$ABTS\;radical\;scavenging\;activity=1-\frac{{ABS}_{sample}}{{ABS}_{control}}.$$

#### 3.3.2. DPPH Radical Scavenging Activity

The DPPH radical scavenging assay method was adapted based on its use in reference number [38]. A total of 0.05 mL of a sample was added to 2.5 mL of a methanolic solution of DPPH (absorbance was approx. 1.2). The samples were incubated in the dark at room temperature for 30 min. Absorbance of solutions was measured at λ = 517 nm against water as a reference. Percentage of antioxidant activity was compared against the positive control, a standard solution of ascorbic acid, and calculated using the following equation [39]:

(2) $$DPPH\;radical\;scavenging\;activity\;(\%)=1-\frac{{ABS}_{sample}}{{ABS}_{control}}.$$

### 3.4. Analysis of Antibacterial Activity and Antibacterial Mechanism of QC NPs

#### 3.4.1. Culture Conditions of Strains

The activated *E. coli* was diluted in a nutrient broth medium to an optical density of 0.5 at a wavelength of 600 nm (OD 600 nm), corresponding to approximately 10^9^ CFU/mL. Subsequently, the *E. coli* cell culture was prepared by mixing *E. coli* with QC NPs, quercetin, and copper ions, respectively, and incubated for 3 h at 37 °C. Additionally, the *S. aureus* cell culture was prepared in accordance with the aforementioned methodology.

#### 3.4.2. Antibacterial Activity Analysis

The antibacterial activity of QC NPs was analyzed using the plate counting method. Serial dilutions of the *E. coli* cell culture (10^6^ CFU/mL) and *S. aureus* cell culture (10^6^ CFU/mL) were prepared and an aliquot was plated onto TTC nutrient agar plates. The plates were then incubated for 24 h at 37 °C, after which the number of colonies was counted.

#### 3.4.3. Field Emission Scanning Electron Microscopy (FESEM) Observation

The FESEM observation method was adapted based on its use in reference number [40]. The *E. coli* cell culture (10^7^ CFU/mL) and *S. aureus* cell culture (10^7^ CFU/mL) were collected (6000× *g*, 5 min) and fixed overnight at 4 °C with 2.5% (*v*/*v*) glutaraldehyde. The samples were dehydrated with graded ethanol (15, 30, 45, 60, 75, 90, and 100%). Finally, the samples were dried and sprayed with gold for observation by FESEM (MIRA, TESCAN, Brno, The Czech Republic).

#### 3.4.4. ROS Content Analysis and Flow Cytometry Analysis

To determine the burst of intracellular ROS induced by QC NPs in bacteria, 2′,7′-dichlorofluorescin diacetate (DCFH-DA) was applied. A reactive oxygen species assay kit (Beyotime, Shanghai, China) was applied to measure ROS as follows: DCFH-DA at a final concentration of 10 μmol L^−1^ was injected into the *E. coli* cell culture (10^6^ CFU/mL) and *S. aureus* cell culture (10^6^ CFU/mL), incubated in the dark for 2 h, and analyzed by flow cytometry (CytoFLEX, Beckman, Indianapolis, IN, USA). The data were analyzed using FlowJo_v10.8.1 software [41,42].

#### 3.4.5. Nucleic Acid and Protein Leakage Analysis

The method used for nucleic acid and protein leakage analysis was that of reference [43]. The *E. coli* cell suspension (10^6^ CFU/mL)- and *S. aureus* cell suspension (10^6^ CFU/mL)-treated samples (QC NPs, quercetin, and copper) were incubated at 37 °C in intervals of 0, 30, 60, 90, 120, 150, and 180 min. Afterward, they were pelleted by centrifugation (10,000 rpm, 4 °C, 10 min) and absorbance of the cell-free supernatant was measured at 260 nm and 280 nm on a microplate reader (Epoch, BioTek, Oviedo, FL, USA) to determine the leakage of nucleic acid and protein.

#### 3.4.6. Biofilm Degradation Activity Analysis

Biofilm degradation activity analysis of QC NPs was optimized based on the methods reported in references [44,45]. The *E. coli* cell culture (10^7^ CFU/mL) and *S. aureus* cell culture (10^7^ CFU/mL) were added separately into 48-well flat-bottomed microplates, followed by incubation at 37 °C for 72 h, where the medium was changed every 24 h during the culture process. Once the incubation period had elapsed, the contents of each well were removed and washed three times with PBS in order to remove any residual bacteria. The biofilm in the wells was fixed with 4% paraformaldehyde for a period of 15 min. Following the removal of the fixative, the biofilm was stained with 0.2% crystal violet for a period of 30 min. Thereafter, the microplates were rinsed with PBS to remove any excess staining and 200 μL of 33% acetic acid was added to each well. The optical density (OD) of each well was then measured at 570 nm using a microplate reader.

### 3.5. Biosafety Analysis

#### 3.5.1. Cytotoxicity Assay

The cytotoxicity assay of the QC NPs was optimized based on the methods reported in reference [46]. The concentration of L929 cells was adjusted to 10^4^ cells/mL and 100 μL of cell suspension was transferred to a 96-well plate and incubated at 37 °C in a CO₂ incubator. The next day, following the adhesion of cells, varying concentrations of QC NPs were added. Following a 24-h period, CCK-8 assay solution was added and incubated at 37 °C for 30 min. The OD values were then measured at 450 nm.

#### 3.5.2. Hemolysis Rates Assay

The hemolysis rates assay of the QC NPs was optimized based on the methods reported in reference [47]. A 5% mouse erythrocyte solution was combined with an equal volume of the sample and incubated for one hour at 37 °C. Following this, the sample was subjected to centrifugation at 1000 rpm for 10 min, after which the absorbance of the supernatant was measured at 540 nm using a microplate reader.

### 3.6. Application to Shine Muscat Grapes

#### 3.6.1. Coating Treatment of Grapes

Fresh *Shine Muscat* grapes were carefully selected and checked for mechanical defects and pests, then soaked in 3% sodium alginate (SA) solution [48]. The grapes in the control group were coated with water only, while the grapes in the positive group were coated with potassium sorbate (0.02% potassium sorbate in water) only. The sample grapes were classified into the SA group and the SA + QC NP (3% SA + 0.1 mg/mL QC NPs) group. Subsequently, the grapes were dried in a ventilated room and stored at 25 °C. Grapes in the different groups were recorded with a camera every 2 days.

#### 3.6.2. Weight Loss

The grapes were weighed every 4 days. The following equation was employed to calculate the weight loss of the grapes:

(3) $$Weight\;Loss\;(\%)=\frac{\left(W_{1}-W_{0}\right)}{W_{0}},$$

where W_0_ is the initial weight and W_1_ is the weight during storage.

#### 3.6.3. Firmness

The firmness of the grapes in the different groups were measured using a texture analyzer (TA. TOUCH, Bosin Tech, Shanghai, China).

#### 3.6.4. Titratable Acid (TA) Content

The titratable acid content assay was conducted according to reference [49]. A total of 2 g of grape homogenate was placed in a 25 mL volumetric flask containing distilled water. After standing for 30 min, the volumetric flask was filtered and 20 mL of the filtrate was taken and titrated with 0.01 mol/L NaOH using 1% phenolphthalein solution as an indicator. The volume of NaOH consumed was recorded when the solution turned pink and remained unchanged for 30 s. Three measurements were taken to calculate the average value. The following equation was employed to calculate the TA Content of the grapes:

(4) $$TA\;\left(\%\;citric\;acid\right)=\frac{V_{2}\times C_{2}\times V_{0}\times 0.064}{m\times V_{1}}$$

where V_2_ is the volume of NaOH solution consumed by titration, C_2_ is the concentration of NaOH solution, 0.064 is the conversion factor of citric acid, V_0_ is the volume of diluted grape homogenate, V_1_ is the volume of grape homogenate used for titration, and m is the weight of the sample.

### 3.7. Statistical Analysis

The experiment was conducted in triplicate and the data were presented as the mean ± standard deviation (SD). Statistical analysis was performed using Origin 2021, GraphPad Prism 8, and SPSS Statistics 24.0.

## 4. Conclusions

This study successfully prepared quercetin/copper nanoparticles (QC NPs) via self-assembly, yielding monodisperse nanoparticles (79.4 ± 3.2 nm) with high colloidal stability (zeta potential: −34.00 ± 4.98 mV). The QC NPs demonstrated potent dual functionality, exhibiting concentration-dependent antioxidant activity (ABTS: EC50 = 0.3814 mg/mL; DPPH: EC50 = 0.0219 mg/mL) and complete bactericidal efficacy (99.99% reduction) against *E. coli* and *S. aureus* at 0.05 mg/mL. Mechanistic studies revealed that QC NPs induced bacterial membrane disruption, intracellular ROS accumulation, and subsequent leakage of nucleic acids and proteins. Biosafety assessments confirmed low cytotoxicity (>95% cell proliferation rate at ≤0.4 mg/mL) and biocompatibility (<5% hemolysis rate at ≤0.1 mg/mL).

When applied as a sodium alginate-based coating, QC NPs + SA significantly reduced weight loss (8.08% vs. 10.40% in controls), preserved firmness, and maintained titratable acidity in *Shine Muscat* grapes, extending shelf life by up to 11 days. These findings highlight the QC NPs + SA coating as an eco-friendly, multifunctional preservative in postharvest fruit protection, combining antioxidant, antimicrobial, and moisture-barrier properties. Future research should focus on scaling production and exploring broader agricultural applications.

## Data Availability

Data will be made available on request.
